# Prediction of Residual Fragments After Flexible Ureteroscopic Stone Management: A Critical Evaluation Based on Patient- and Stone-Related Parameters

**DOI:** 10.3390/jcm14134739

**Published:** 2025-07-04

**Authors:** Hikmet Yaşar, Alper Aşik, Erhan Erdoğan, Göksu Sarica, Abdullah Aydin, Salih Yildirim, Kemal Sarica

**Affiliations:** 1 Department of Urology, Sancaktepe Sehit Prof. Dr. Ilhan Varank Research and Training Hospital, 34785 Istanbul, Turkey; alperasik001@hotmail.com (A.A.); erhandr@hotmail.com (E.E.); abdullahaydn6363@gmail.com (A.A.); yldrmsalih7@gmail.com (S.Y.); saricakemal@gmail.com (K.S.); 2 Medical School, Biruni University, 34015 Istanbul, Turkey; goksusarica16@gmail.com; 3 Department of Urology, Medical School, Biruni University, 34015 Istanbul, Turkey

**Keywords:** retrograde intrarenal surgery, residual fragments, hydronephrosis, stone size

## Abstract

**Aim**: This study aimed to evaluate the potential impact of stone characteristics, patient factors, and upper tract anatomical parameters in the prediction of residual fragments (RFs) following the flexible ureteroscopic (fURS) management of renal stones. **Patients and Methods**: Between June 2023 and July 2024, a total of 104 cases underwent fURS for the minimally invasive management of medium-sized renal stones (10–25 mm), and 28 cases presenting with RFs 3 months after these procedures were included for further evaluation. In addition to the assessment of patient-related factors, non-contrast computed tomography (NCCT) was performed in all cases in an attempt to assess specific stone characteristics and upper tract anatomical parameters in detail during the 3-month follow-up period. **Results**: An evaluation of our findings indicated that, among the evaluated parameters, a higher degree of hydronephrosis (>Grade 2), a larger stone size (>15 mm), and the presence of multiple stones were found to affect the presence of RFs significantly (*p* = 0.020, *p* = 0.012, and *p* = 0.040, respectively). On the other hand, although the analysis of other parameters such as patient gender, stone side, stone hardness, and the use of an access sheath with univariate regression demonstrated potential correlations, none of these parameters demonstrated a significant impact when analyzed using backward logistic regression. Logistic regression revealed that Grade 2 hydronephrosis (OR = 18.3, *p* = 0.020), stone size > 15 mm (OR = 7.0, *p* = 0.012), and multiple stones (OR = 3.7, *p* = 0.040) significantly increased the risk of residual fragments following fURS. **Conclusions**: In light of our findings and published data, we can conclude that endourologists should consider the likelihood of RFs’ presence after successful stone disintegration with fURS. A detailed evaluation of the relevant factors revealed that patients with larger stones, higher degrees of hydronephrosis, and multiple calculi may carry the risk of residual fragments after these procedures. Thus, the utilization of such reliable predictive parameters may aid in selecting optimal stone removal strategies and planning subsequent interventions in the rational management of RFs.

## 1. Introduction

As a result of significant technological advancements and rapidly evolving treatment concepts over the last three decades, the management of urinary stones has undergone a dramatic transformation. Open surgery is now applied in only a small percentage of cases with symptomatic stones. Among the available techniques for the disintegration and removal of urinary calculi, extracorporeal shock wave lithotripsy (ESWL), flexible ureteroscopy (fURS), and percutaneous nephrolithotomy (PCNL) are the most commonly utilized methods, rendering patients stone-free in current clinical practice [1,2,3,4,5,6].

Advancements in ureteroscope technology, the application of holmium/yttrium–aluminum–garnet (Ho:YAG) lasers, and the highly effective use of accessory instruments have led urologists to apply fURS as a widely accepted modality in the minimally invasive management of small- to medium-sized renal stones (10–20 mm). Despite its relatively more invasive nature compared to ESWL, fURS constitutes a considerably less invasive alternative to PCNL with safe and effective applications, leading to the rapid adoption of this technique worldwide [7,8]. Supporting this trend, the European Association of Urology (EAU) and the American Urological Association (AUA) urolithiasis guidelines recommend ESWL and fURS as equally effective treatment modalities in the minimally invasive and effective removal of kidney stones smaller than 20 mm [5,9,10].

Although published data so far have demonstrated that the application of fURS in such stones is associated with stone-free rates (SFR) ranging from 73.6% to 94.1%, residual fragments (RFs) have been detected in 5.9% to 26.4% of cases undergoing these procedures. In most of the cited studies, stone-free status is defined as the absence of residual fragments larger than 2 to 4 mm, with ≤4 mm being the most commonly accepted cutoff. Accordingly, RFs refer to stone remnants exceeding these size thresholds, typically located in the calyces or renal pelvis [11,12,13,14,15,16]. In the light of the ultimate goal concerning the achievement of completely stone-free (SF) status in a single session, the presence of RF is considered an “unsuccessful outcome” by all endourologists, which can complicate the follow-up process for both patients and clinicians. Related to this issue, several scoring systems and nomograms have been proposed in recent years to predict SFR after fURS [17,18]. While some of these tools incorporate a combination of radiological and clinical variables, their predictive accuracy remains variable, and external validations have shown inconsistent results [11]. The use of a ureteral access sheath (UAS) has become a standard adjunct in many fURS procedures, aiming to facilitate instrument insertion, improve visibility, and reduce intrarenal pressure, all of which may influence procedural success and the likelihood of residual fragments.

Accumulated evidence so far has revealed that residual fragments following any stone removal procedure may cause pain, infection, and obstruction during the follow-up period, which may potentially affect the life quality of the patients undergoing these procedures. Furthermore, secondary procedures required for the removal of such fragments may increase the morbidity risk and add to the overall healthcare costs. Taking all these abovementioned challenges into account, identification of the stone- and patient-related factors which may play a critical role in the reliable prediction of the presence as well as the size of RFs will be highly beneficial in routine clinical practice [18,19].

In this study, we primarily aimed to outline the stone-, patient-, and urinary-tract-anatomy-related factors in the effective prediction of the presence and size of residual fragments following fURS procedures performed for medium-sized (10–15 mm) stones.

### Patients and Methods

Between June 2023 and July 2024, departmental file records of all cases (*n* : 104) undergoing fURS for renal stones were retrospectively evaluated in detail. Adult cases (>18 years) with renal stones measuring 10–25 mm were included, while those with renal abnormalities, preoperative ureteral stents, ureteral stenosis, or intraoperative complications were excluded. After applying the inclusion criteria, a total of 104 cases were included in the study. Patients with a history of upper tract urothelial carcinoma (UTUC) or pregnancy at the time of diagnosis or treatment were excluded from the study.

Preoperative evaluation included detailed anamnesis, comprehensive biochemical testing, and radiological assessments. Radiological examinations included kidney–ureter–bladder (KUB) radiography, urinary ultrasonography, and non-contrast computed tomography (NCCT) to evaluate stone-related factors and the renal collecting system’s status. Stone size and residual fragments were assessed using NCCT images.

Patients with positive preoperative urine cultures received targeted antibiotic therapy based on culture and sensitivity results. Only those whose follow-up control cultures confirmed sterile urine were included in the procedure. Thus, all cases had sterile urine at the time of surgery. For prophylaxis, all patients received a single dose of third-generation cephalosporin immediately before surgery. Patients were considered stone-free if no stone fragments or fragments < 4 mm in diameter were present.

Based on radiological evaluations, primarily using NCCT, performed three months postoperatively, 28 patients with residual stones were ultimately included in this study. Pre- and postoperative patient-related and radiological parameters were comparatively analyzed to identify potential factors influencing the presence of RF.

All surgeries were performed under general anesthesia in the lithotomy position. Under fluoroscopic guidance, a 0.038 Fr guidewire was inserted into the renal pelvis using a 7.5 Fr semi-rigid ureteroscope (Wolf). Retrograde pyelography was performed to evaluate the pelvicalyceal system. A classical 9.5/11.5 Fr ureteral access sheath (Cook Medical, Bloomington, IN, USA) was placed over the guidewire under fluoroscopy. The collecting system was accessed using a 7.5 Fr fiber optic flexible ureteroscope (Storz FLEX-X2, Storz & Bickel, Tuttlingen, Germany) passed through the UAS. Stones were fragmented using a holmium laser (273 μm fiber), and fragments > 3 mm in diameter were retrieved using a nitinol basket (ZeroTip™, Cook Urological Inc., Spencer, IN, USA). A 4.8 Fr JJ stent was placed postoperatively and removed after two weeks.

All procedures were performed by two senior endourologists, each with more than five years of clinical and surgical experience in retrograde intrarenal surgery (fURS). Hydronephrosis grading was standardized and conducted based on non-contrast computed tomography (NCCT) images, following commonly accepted radiological criteria (Grade 0: no dilation; Grade 1: mild; Grade 2: moderate) [13]. Postoperative evaluation of residual fragments was performed using NCCT at three months. All radiological assessments were conducted by a single experienced radiologist who was blinded to the patients’ clinical data and surgical outcomes, thus minimizing assessment bias and eliminating inter-observer variability.

SPSS v.22.0 software (SPSS Inc., Chicago, IL, USA) was used for statistical analyses. Means and standard deviations were reported for continuous variables. The Spearman correlation test was applied to analyze associations between groups, and the Mann–Whitney U test was used for categorical variables. The odds ratio and corresponding 95% confidence intervals were calculated to assess the significance of impact. A *p*-value of <0.05 was considered statistically significant in all analyses.

Ethical approval for this retrospective study was obtained from the Ethics Committee of Sancaktepe Şehit Prof. Dr. İlhan Varank Training and Research Hospital (Approval No: E-46059653-050.99-256509952; Date: 9 October 2024). The requirement for informed consent was waived due to the retrospective nature of the study.

## 2. Results

The mean age of the study cohort was 47.06 ± 12.74 years (range: 20–75 years), with 36.54% of cases being female and 63.46% being male. The mean stone size was less than 15 mm (10–15 mm) in 69.23% of cases, while 30.77% had stones larger than 15 mm (16–25 mm). Regarding the number of stones, 76.92% of cases presented with a single stone, whereas 23.08% had multiple stones at initial evaluation.

Assessment of hydronephrosis status showed that 28.85% of patients had no hydronephrosis, 42.31% had Grade 1 hydronephrosis, and 28.85% had Grade 2 hydronephrosis. The operative side was the right in 51.92% of cases and the left in 48.08%. The calyceal distribution of stones revealed that 46.15% were located in the lower calyx, while the remaining 53.85% involved other calyces or the renal pelvis.

Operative parameters showed that a ureteral access sheath (UAS) was used in 75% of cases, while no UAS was employed in 25%. Stone hardness (HU value) was <1000 in 55.77% of cases and ≥1000 in 44.23%. Postoperative NCCT evaluation during the three-month follow-up revealed no residual fragments in 73.08% of cases, while 26.92% had residual fragments (Table 1).

Univariate analysis demonstrated that hydronephrosis Grade 1, UAS usage, and stone hardness > 1000 HU had no significant impact on RF presence (*p* > 0.05). Conversely, higher degrees of hydronephrosis (Grade > 1), multiple stones, and larger stone size (>15 mm) were significantly associated with RF presence (*p* < 0.05).

Logistic regression analysis of these three significant parameters revealed an explanatory coefficient of 82.7%, with a sensitivity of 57.1% and specificity of 92.1%. Backward (conditional) logistic regression further confirmed the significance of higher hydronephrosis (Grade 2), stone size > 15 mm, and multiple stones (*p* = 0.020, *p* = 0.012, *p* = 0.040, respectively). Despite univariate regression suggesting correlations with other parameters, they were not significant in the logistic model (*p* > 0.05).

The risk of RF presence after fURS was found to be 18 times higher in patients with higher hydronephrosis (OR = 18.312, 95% CI: 1.569–213.710), 7 times higher in those with stones > 15 mm (OR = 6.953, 95% CI: 1.537–31.450), and 3.7 times higher in patients with multiple stones (OR = 3.700, 95% CI: 1.690–19.841) (Table 2).

## 3. Discussion

The management of symptomatic renal stones has undergone significant changes due to advances in instrument technology, evolving treatment concepts, and increasing clinical experience. While percutaneous nephrolithotomy (PCNL) is the preferred modality for large or complex stones (>20 mm), flexible ureteroscopy (fURS) has become a commonly applied technique worldwide for medium-sized stones (10–20 mm) due to its lower complication rates and high success rates when performed by experienced practitioners [20,21,22]. Despite its widespread use and favorable outcomes, fURS carries the risk of certain complications, among which the presence of residual fragments (RFs) represents a significant problem following the application of the technique.

Minimally invasive procedures aim to achieve complete stone-free (SF) status in a single session, leaving no RFs if possible. However, certain factors, including stone size, number, location, density, patient-related variables (e.g., BMI, anatomy of the collecting system), and surgeon’s experience, can contribute to the presence of RFs, particularly following ESWL and fURS, where some fragments may be left for spontaneous passage. The incidence of RFs after fURS varies between 5.9% and 26.4% in published reports [23,24,25].

Considering the potential complications associated with RFs, such as infection, pain, and obstruction, identifying the factors 0that may predict RF presence seems to be highly critical. Although scoring systems have been developed to estimate RF risk after fURS, their outcomes are often contradictory and insufficient for reliable clinical utility. Moreover, limited studies so far have evaluated the factors contributing to RF formation in a comprehensive manner.

Several nephrolithometric scoring systems have been developed to predict stone-free status following retrograde intrarenal surgery, including the Resorlu–Unsal Stone Score (RUSS), the R.I.R.S. score, and other nomogram-based models [11,12,14,15]. While these models incorporate various clinical and radiological parameters such as stone size, number, density, and pelvicalyceal anatomy, their predictive accuracy remains variable. For instance, Selmi et al. and Erbin et al. demonstrated that although these systems may aid in clinical decision-making, their external validation results have shown inconsistent predictive performance [11,12]. In our study, we did not apply a scoring system per se; however, key parameters commonly included in these models—such as stone size, hydronephrosis grade, and multiplicity—were found to significantly correlate with residual fragment presence, aligning with findings from previous nephrolithometric studies.

Moreover, limited studies so far have evaluated the factors contributing to RF formation in a comprehensive manner, with most focusing on individual parameters or selective scoring systems [11,12].

The published data suggest that larger stone size and higher Hounsfield unit (HU) values are associated with lower SF rates and an increased need for auxiliary procedures after fURS [26]. For instance, Koç and Yıldırım demonstrated a correlation between preoperative stone size, HU values, and the size of RF after the procedure [27,28]. Additionally, studies by Reşorlu, Jesseb, Danilovic, and Dresner highlighted that as a critical anatomical parameter, lower infundibulopelvic angle (IPA) correlates with decreased SF rates for lower pole stones treated with fURS [29,30,31,32]. Karim et al. further confirmed this in their review, showing that reduced IPA is linked to increased RF rates [33].

The S.T.O.N.E. scoring system (size, topography, obstruction, number, and evaluation of HU), as proposed by Molina and Ito et al., indicates a negative correlation between SF rates and the severity of hydronephrosis in the involved kidney [34,35]. Yıldırım and Tanık observed that stone hardness does not significantly affect SF rates [31,36]. A meta-analysis by Özman et al. also identified stone number and location as factors influencing SF rates, particularly for lower calyx stones [32]. Finally, while Altay and Turna could not show any positive correlation between the use of anticoagulants and SFRs after fURS, Yıldırım et al. demonstrated that the use of anticoagulants could reduce SFRs following this procedure [33,34]. These findings are also supported by recent work by Amparore et al. [36], who demonstrated the clinical relevance of nephrolithometric scoring systems in predicting outcomes of RIRS, though they emphasized the need for further external validation.

In this study, we assessed the impact of stone-, patient-, and upper-tract-anatomy-related parameters on the presence and size of RFs after fURS procedures. Our findings reveal that a higher hydronephrosis degree (Grade 2), larger stone size (>15 mm), and multiple stones do significantly influence the occurrence of RFs (*p* = 0.020, *p* = 0.012, *p* = 0.040, respectively). Related to this issue, our results reveal that although there were potential correlations between RF presence and other parameters (e.g., patient gender, stone side, stone hardness, UAS use) in univariate regression analysis, these correlations were not found to be statistically significant in backward (conditional) logistic regression analysis (*p* > 0.05).

Although variables such as patient gender, stone side, stone hardness (HU), and UAS usage showed potential associations in univariate analysis, they did not retain statistical significance in multivariate regression. This may be due to the stronger predictive value of dominant variables such as stone size and hydronephrosis grade, or possible confounding effects within the dataset. The lack of significance for HU, for example, may reflect the overriding influence of stone location or number on fragment clearance, as suggested in previous studies [13,22,23].

From a clinical standpoint, our findings highlight the value of preoperative evaluation of stone characteristics and renal anatomy in predicting residual fragments. Identifying high-risk patients—those with multiple stones, stones > 15 mm, or Grade 2 hydronephrosis—can aid in surgical planning, patient counseling, and possibly the development of simplified risk prediction tools [11,12,32].

In light of our current findings and data reported in the literature so far, it is clear that, in order to achieve a completely SF status, endourologists should focus on the most suitable treatment option during the decision-making phase. All predictive parameters need to be considered, and this issue is especially critical for certain procedures where some fragments are left for spontaneous passage under close follow-up. Using preoperative stone- and patient-related parameters to predict RF likelihood can aid in this important planning process. Based on our findings, by using these parameters, endourologists can also inform patients about the risk of RF presence, potential complications, and the need for additional procedures.

This study has several limitations that should be acknowledged. First, its retrospective, single-center design may introduce selection bias and limit the generalizability of the findings. Second, although all surgeries were performed by experienced endourologists following a standardized protocol, subtle intraoperative variations could have influenced outcomes. Third, although several parameters were included in the multivariable model, the relatively low number of events (*n* = 28) may have increased the risk of model overfitting. To reduce this risk, we applied a backward stepwise (conditional) logistic regression approach to identify the most relevant predictors. Fourth, stone composition analysis was not uniformly available for all cases, which may have affected the assessment of fragmentation and residual fragment formation. Lastly, the three-month follow-up period may not have captured delayed stone clearance or recurrence in some patients. Future prospective, multicenter studies with longer follow-up and standardized stone analysis are warranted to validate our findings.

## 4. Conclusions

In light of our results and the limited data in the literature, endourologists should be cautious about RFs’ presence and related complications following fURS, particularly in cases with larger stones, higher hydronephrosis degrees, and multiple calculi. This approach will enable physicians to perform more rational decision-making for stone removal and to prepare for potential subsequent procedures to manage RFs.

## Figures and Tables

**Table 1:** Evaluation of demographic findings and stone-related parameters.

Parameter	Categories	*n* (%)	Median (Min–Max)
Age			46 (20–75)
Gender	Female	38 (36.54)	
	Male	66 (63.46)	
Hydronephrosis	None	30 (28.85)	
	Grade 1	44 (42.31)	
	Grade 2	30 (28.85)	
Lateralization	Right	54 (51.92)	
	Left	50 (48.08)	
Stone Localization	Lower calyx	48 (46.15)	
	Pelvis	56 (53.85)	
Use of Access Sheath	No	26 (25.00)	
	Yes	78(75.00)	
Stone Multiplicity	Single	80 (76.92)	
	Multiple	24 (23.08)	
Stone Size (mm)	≤15	72 (69.23)	14 (10–25)
	>15	32 (30.77)	
Stone Density (HU)	≤1000	58 (55.77)	1000 (500–1600)
	>1000	46 (44.23)	

HU, Hounsfield unit.

**Table 2:** Logistic regression analysis of risk factors affecting residual stone formation.

Variable	Univariate *p* Value	OR (95% CI)	Multivariate *p* Value	OR (95% CI)
Hydronephrosis (Grade 1)	0.146	5.250 (0.562–49.080)	0.096	7.528 (0.698–81.152)
Hydronephrosis (Grade 2)	0.030	12.250 (1.268–118.361)	0.020	18.312 (1.569–213.710)
Use of Access Sheath (None)	0.719	0.764 (0.176–3.310)	–	–
Stone Multiplicity (Multiple)	0.009	6.600 (1.615–26.977)	0.040	3.700 (1.690–19.841)
Stone Size > 15 mm	0.016	5.000 (1.343–18.620)	0.012	6.953 (1.537–31.450)
Stone Density > 1000 HU	0.455	0.617 (0.174–2.187)	–	–

## Data Availability

The data presented in this study are available within the hospital’s electronic medical record system and are accessible upon reasonable request.

## References

[1] Scales C.D., Smith A.C., Hanley J.M., Saigal C.S., Urologic Diseases in America Project (2020). Prevalence of kidney stones in the United States. Eur. Urol..

[2] Ordon M., Urbach D., Mamdani M., Saskin R., D’AHoney R.J., Pace K.T. (2014). The surgical management of kidney stone disease: A population based time series analysis. J. Urol..

[3] Chew B.H., Brotherhood H.L., Sur R.L., Wang J., Knudsen B.E., Lange D., Ordon M., Paterson R.F., Pautler S.E., Razvi H. (2021). Surgical trends in the management of urolithiasis: A decade in review. J. Urol..

[4] Bhanot R., Jones P., Somani B.K. (2021). Minimally invasive surgery for the treatment of ureteric stones—State-of-the-art review. Res. Rep. Urol..

[5] Van Cleynenbreugel B., Akand M., Kılıç ö. (2017). Retrograde intrarenal surgery for renal stones—Part 2. Turk. J. Urol..

[6] Türk C., Skolarikos A., Neisius A., Petřík A., Tailly T., Thomas K., Davis N.F., Geraghty R., Somani B.K., Seitz C. (2023). EAU Guidelines on Urolithiasis 2023. Eur. Urol..

[7] Scales C.D., Lai J.C., Dick A.W., Hanley J.M., van Meijgaard J., Setodji C.M., Saigal C.S., Urologic Diseases in America Project (2014). Comparative effectiveness of shock wave lithotripsy and ureteroscopy for treating patients with kidney stones. JAMA Surg..

[8] Zhong W., Zeng G., Wu K., Chen W., Wu W., Desai J., Zhao Z., Peng L., Ek y. (2020). Efficacy and safety of minimally invasive PCNL versus open surgery: A multicenter prospective study. World J. Urol..

[9] Sfoungaristos S., Gofrit O.N., Mykoniatis I., Landau E.H., Katafigiotis I., Pode D., Constantinides C.A., Duvdevani M. (2016). External validation of Resorlu-Unsal stone score as predictor of outcomes after retrograde intrarenal surgery. Int. Urol. Nephrol..

[10] Wang C., Wang S., Wang X., Lu J. (2021). External validation of the R.I.R.S. scoring system to predict stone-free rate after retrograde intrarenal surgery. BMC Urol..

[11] Ma Y., Jian Z., Xiang L., Zhou L., Jin X., Luo D., Li H., Wang K.J. (2022). Development of a novel predictive model for a successful stone removal after flexible ureteroscopic lithotripsy based on ipsilateral renal function: A single-centre, retrospective cohort study in China. BMJ Open.

[12] Kim J.W., Chae J.Y., Kim J.W., Oh M.M., Park H.S., Moon D.U.G., Yoon C.Y. (2013). Computed tomography-based novel prediction model for the stone-free rate of ureteroscopic lithotripsy. Urolithiasis.

[13] Selmi V., Sari S., Oztekin U., Caniklioglu M., Isikay L. (2021). External Validation and Comparison of Nephrolithometric Scoring Systems Predicting Outcomes of Retrograde Intrarenal Surgery. J. Endourol..

[14] Erbin A., Tepeler A., Buldu I., Ozdemir H., Tosun M., Binbay M. (2016). External Comparison of Recent Predictive Nomograms for Stone-Free Rate Using Retrograde Flexible Ureteroscopy with Laser Lithotripsy. J. Endourol..

[15] Ergani B., Ozbilen M.H., Yalcın M.Y., Boyacıoglu H., Ilbey Y.O. (2021). The effect of hydronephrosis grade on stone-free rate in retrograde intrarenal stone surgery with flexible ureterorenoscopy. Am. J. Clin. Exp. Urol..

[16] Resorlu B., Unsal A., Gulec H., Oztuna D. (2012). A new scoring system for predicting stone-free rate after retrograde intrarenal surgery: The “resorlu-unsal stone score”. Urology.

[17] Xiao Y., Li D., Chen L., Xu Y., Zhang D., Shao Y., Lu J. (2017). The R.I.R.S. scoring system: An innovative scoring system for predicting stone-free rate following retrograde intrarenal surgery. BMC Urol..

[18] De S., Autorino R., Kim F.J., Zargar H., Laydner H., Balsamo R., Torricelli F.C., Di Palma C., Molina W.R., Monga M. (2015). Percutaneous nephrolithotomy versus retrograde intrarenal surgery: A systematic review and meta-analysis. Eur. Urol..

[19] Breda A., Ogunyemi O., Leppert J.T., Schulam P.G. (2009). Flexible ureteroscopy and laser lithotripsy for multiple unilateral intrarenal stones. Eur. Urol..

[20] Rippel C.A., Nikkel L., Lin Y.K., Danawala Z., Olorunnisomo V., Youssef R.F., Pearle M.S., Lotan Y., Raman J.D. (2012). Residual fragments following ureteroscopic lithotripsy: Incidence and predictors on postoperative computerized tomography. J. Urol..

[21] Gu J., Luo S., Jiang L., Hu D., Zhao G., Tang W. (2022). Novel scoring system combined with a virtual reality technique for the preoperative evaluation of the stone-free status after flexible ureteroscopy: The H.L.P.E.S. score. BMC Urol..

[22] Adiga P., D’souza R.C.C., Nandakishore B., Shetty M., Adiga P., Dsouza R. (2024). Assessment of Factors Responsible for Stone-Free Status After Retrograde Intrarenal Surgery. Cureus.

[23] Uslu M., Yıldırım Ü., Ezer M., Erihan I.B., Sarıca K. (2023). Residual fragment size following retrograde intrarenal surgery: A critical evaluation of related variables. Urolithiasis.

[24] Koc E., Kamaci D., Gok B., Bedir F., Metin B.C., Atmaca A.F. (2020). Does the renal parenchymal thickness affect the efficacy of the retrograde intrarenal surgery? A prospective cohort study. Urolithiasis.

[25] Resorlu B., Oguz U., Resorlu E.B., Oztuna D., Unsal A. (2012). The impact of pelvicaliceal anatomy on the success of retrograde intrarenal surgery in patients with lower pole renal stones. Urology.

[26] Jessen J.P., Honeck P., Knoll T., Wendt-Nordahl G. (2014). Flexible ureterorenoscopy for lower pole stones: Influence of the collecting system’s anatomy. J. Endourol..

[27] Danilovic A., Cavalanti A., Rocha B.A., Traxer O., Torricelli F.C.M., Marchini G.S., Mazzucchi E., Srougi M. (2018). Assessment of Residual Stone Fragments After Retrograde Intrarenal Surgery. J. Endourol..

[28] Dresner S.L., Iremashvili V., Best S.L., Hedican S.P., Nakada S.Y. (2020). Influence of Lower Pole Infundibulopelvic Angle on Success of Retrograde Flexible Ureteroscopy and Laser Lithotripsy for the Treatment of Renal Stones. J. Endourol..

[29] Karim S.S., Hanna L., Geraghty R., Somani B.K. (2020). Role of pelvicalyceal anatomy in the outcomes of retrograde intrarenal surgery (RIRS) for lower pole stones: Outcomes with a systematic review of literature. Urolithiasis.

[30] Molina W.R., Kim F.J., Spendlove J., Pompeo A.S., Sillau S., Sehrt D.E. (2014). The S.T.O.N.E. Score: A new assessment tool to predict stone free rates in ureteroscopy from pre-operative radiological features. Int. Braz. J. Urol. Off. J. Braz. Soc. Urol..

[31] Ito H., Sakamaki K., Kawahara T., Terao H., Yasuda K., Kuroda S., Yao M., Kubota Y., Matsuzaki J. (2015). Development and internal validation of a nomogram for predicting stone-free status after flexible ureteroscopy for renal stones. BJU Int..

[32] Tanik S., Zengin K., Albayrak S., Atar M., Imamoglu M.A., Bakirtas H., Gurdal M. (2015). The effectiveness of flexible ureterorenoscopy for opaque and non-opaque renal stones. Urol. J..

[33] Yildirim Ü., Ezer M., Uslu M., Güzel R., Sarica K. (2023). Can additional variables be used to predict stone-free rates following retrograde intrarenal surgery? Anticoagulants and parenchyma thickness: A detailed examination. Urolithiasis.

[34] Özman O., Akgül H.M., Başataç C., Sancak E.B., Çınar Ö., Çakır H., Yazıcı C.M., Akpınar H., Önal B., RIRSearch Study Group (2022). Recent scoring systems predicting stone-free status after retrograde intrarenal surgery; a systematic review and meta-analysis. Central Eur. J. Urol..

[35] Turna B., Stein R.J., Smaldone M.C., Santos B.R., Kefer J.C., Jackman S.V., Averch T.D., Desai M.M. (2008). Safety and efficacy of flexible ureterorenoscopy and holmium:YAG lithotripsy for ıntrarenal stones in anticoagulated cases. J. Urol..

[36] Altay B., Erkurt B., Albayrak S. (2017). A review study to evaluate holmium:YAG laser lithotripsy with flexible ureteroscopy in patients on ongoing oral anticoagulant therapy. Lasers Med. Sci..

